# Supplementary material for: Flax-Derived Carbon: A Highly Durable Electrode Material for Electrochemical Double-Layer Supercapacitors
Source: Nanomaterials (Basel). 2021 Aug 29;11(9):2229. doi: 10.3390/nano11092229 (PMC8466636; doi:10.3390/nano11092229)
Supplement: Supplementary file 1 [file nanomaterials-11-02229-s001.zip › nanomaterials-1350405-supplementary.pdf]

Supporting Information

Flax-Derived Carbon: A Highly Durable Electrode Material for Electrochemical Double-Layer Supercapacitors

Petr Jakubec [1,*,†], Stanislav Bartusek [2,†], Josef Jan Dvořáček [2], Veronika Šedajová [1,3], Vojtěch Kupka [1] and Michal Otyepka [1,*]

[1] Czech Advanced Technology and Research Institute (CATRIN), Regional Centre of Advanced Technologies and Materials (RCPTM), Palacký University Olomouc, Šlechtitelů 27, 783 71 Olomouc, Czech Republic; veronika.sedajova@upol.cz (V.Š.); vojtech.kupka@upol.cz (V.K.)

[2] Department of Environmental Protection in Industry, Faculty of Materials Science and Technology, VSB- Technical University of Ostrava, 17. Listopadu 2172/15, 708 00 Ostrava-Poruba, Czech Republic;
stanislav.bartusek@vsb.cz (S.B.); jdvoracek@solitaire.cz (J.J.D.)

[3] Department of Physical Chemistry, Faculty of Science, Palacký University, 17. Listopadu 1192/12, 779 00 Olomouc, Czech Republic

* Correspondence: p.jakubec@upol.cz (P.J.); michal.otyepka@upol.cz (M.O.); Tel.: +420-585-634-474 (P.J.); +420-585-634-764 (M.O.)

† These authors contributed equally to this work.

Table S1. Electrochemical performance of various activated carbons prepared from different biomass sources.

Sample Name	Biomass Precursor	ActivatinG agent	Electrolyte	Electrode System	Cs [F/g]	Stability [cycles]	E [Wh/kg]	P [W/kg]	Ref
**C-Flax**	flax	KOH	1 M H_2SO_4	3	500 (0.25 A/g)	—	—	—	this work
**C-Flax**	flax	KOH	1 M H_2SO_4	2	189 (0.5 A/g)	85% (150 000 @ 5 A/g)	6.58	250	this work
**CFC**	flax textile	N/A	0.1 M Na_2SO_4	2	1.56 (0.1 A/g)	100% (10 000 @ 5 A/g)	—	—	[1]
**FF-850**	flax fabric	N/A	1 M H_2SO_4	2	12.7 (2 mV/s)	N/A	—	—	[2]

**CF-850**	cotton fabric	N/A	1 M H_2SO_4	2	21.1 (2 mV/s)	N/A	—	—	2
**CF-CNT**	flax fabric	N/A	6 M KOH	3	34 (1 A/g)	N/A	—	—	3
**KHC**	banana stem	KOH	6 M KOH	3	479.23 (1 mV/s)	72.88% (6000 @ 1 A/g)	—	—	4
**PHC**	banana stem	H_3PO_4	6 M KOH	3	202.11 (2mV/s)	—	—	—	4
**CHC**	corn-cob	N/A	6 M KOH	3	309.81 (2 mV/s)	—	—	—	4
**SHC**	potato starch	N/A	6 M KOH	3	99.9 (2 mV/s)	—	—	—	4
**MC-800**	acacia gum	KOH	6 M KOH	3	272 (1 A/g)	93% (1000 @ 1 A/g)	—	—	5
**MC-800**	acacia gum	KOH	6 M KOH	2	223 (1 A/g)		7.76	500	5
**AC-700-15**	sunflower seed shell	KOH	30% KOH	2	244 (0.25 A/g)	—	4.8	2400	6
**PC4/1-C**	rice husk	$ZnCl_2$	6 M KOH	2	233 (2 A/g)	99% (1000 @ 0.05 A/g)	8.36	N/A	7
**PC4/1-C**	rice husk	$ZnCl_2$	6 M KOH	2	242 (2 mV/s)	—	—	—	7
**N1**	rice husk	NaOH	3 M KCl	2	210 (0.2 mA)	—	—	—	8
**AC-900**	carrageenan	KOH	6 M KOH	3	230 (1 A/g)	95% (1000 @ 1 A/g)	—	—	9
**TWPC**	tea	KOH	6 M KOH	3	332 (1 A/g)	99.4% (100000 @ 20 A/g)	—	—	10
**N-SAC-3**	shaddock peel	KOH	6 M KOH	3	325 (1 A/g)	89.8% (10000 @ 100 mV/s)	—	—	11
**C-750**	waste coffee ground	CO_2	5 M KOH	3	190 (5 mV/s)	92% (2000 @ 1 A/g)	—	—	12
—	firwoods	steam	0.5 M H_2SO_4	3	118 (10 A/g)	—	—	—	13

References

1. He, S.; Chen, W. Application of Biomass-Derived Flexible Carbon Cloth Coated with MnO_2 Nanosheets in Supercapacitors. *J. Power Sources* **2015**, *294*, 150–158.
2. Xiao, P.-W.; Meng, Q.; Zhao, L.; Li, J.-J.; Wei, Z.; Han, B.-H. Biomass-Derived Flexible Porous Carbon Materials and Their Applications in Supercapacitor and Gas Adsorption. *Mater. Des.* **2017**, *129*, 164–172.
3. Zhang, Y.; Mao, T.; Wu, H.; Cheng, L.; Zheng, L. Carbon Nanotubes Grown on Flax Fabric as Hierarchical All-Carbon Flexible Electrodes for Supercapacitors. *Adv. Mater. Interfaces* **2017**, *4* (9), 1601123.

4. Ghosh, S.; Santhosh, R.; Jeniffer, S.; Raghavan, V.; Jacob, G.; Nanaji, K.; Kollu, P.; Jeong, S. K.; Grace, A. N. Natural Biomass Derived Hard Carbon and Activated Carbons as Electrochemical Supercapacitor Electrodes. *Sci. Rep.* **2019**, *9* (1), 16315.
5. Fan, Y.; Liu, P.; Zhu, B.; Chen, S.; Yao, K.; Han, R. Microporous Carbon Derived from Acacia Gum with Tuned Porosity for High-Performance Electrochemical Capacitors. *Int. J. Hydrog. Energy* **2015**, *40* (18), 6188–6196.
6. Li, X.; Xing, W.; Zhuo, S.; Zhou, J.; Li, F.; Qiao, S.-Z.; Lu, G.-Q. Preparation of Capacitor's Electrode from Sunflower Seed Shell. *Bioresour. Technol.* **2011**, *102* (2), 1118–1123.
7. He, X.; Ling, P.; Yu, M.; Wang, X.; Zhang, X.; Zheng, M. Rice Husk-Derived Porous Carbons with High Capacitance by ZnCl2 Activation for Supercapacitors. *Electrochim. Acta* **2013**, *105*, 635–641.
8. Guo, Y.; Qi, J.; Jiang, Y.; Yang, S.; Wang, Z.; Xu, H. Performance of Electrical Double Layer Capacitors with Porous Carbons Derived from Rice Husk. *Mater. Chem. Phys.* **2003**, *80* (3), 704–709.
9. Fan, Y.; Yang, X.; Zhu, B.; Liu, P.-F.; Lu, H.-T. Micro-Mesoporous Carbon Spheres Derived from Carrageenan as Electrode Material for Supercapacitors. *J. Power Sources* **2014**, *268*, 584–590.
10. Khan, A.; Senthil, R. A.; Pan, J.; Osman, S.; Sun, Y.; Shu, X. A New Biomass Derived Rod-like Porous Carbon from Tea-Waste as Inexpensive and Sustainable Energy Material for Advanced Supercapacitor Application. *Electrochim. Acta* **2020**, *335*, 135588.
11. Chen, J.; Lin, Y.; Liu, J.; Wu, D.; Bai, X.; Chen, D.; Li, H. Outstanding Supercapacitor Performance of Nitrogen-Doped Activated Carbon Derived from Shaddock Peel. *J. Energy Storage* **2021**, *39*, 102640.
12. Hossain, R.; Nekouei, R. K.; Mansuri, I.; Sahajwalla, V. In-Situ O/N-Heteroatom Enriched Activated Carbon by Sustainable Thermal Transformation of Waste Coffee Grounds for Supercapacitor Material. *J. Energy Storage* **2021**, *33*, 102113.
13. Wu, F.-C.; Tseng, R.-L.; Hu, C.-C.; Wang, C.-C. Physical and Electrochemical Characterization of Activated Carbons Prepared from Firwoods for Supercapacitors. *J. Power Sources* **2004**, *138* (1–2), 351–359.
